# Allelic Combinations of Soybean Maturity Loci *E1*, *E2*, *E3* and *E4* Result in Diversity of Maturity and Adaptation to Different Latitudes

**DOI:** 10.1371/journal.pone.0106042

**Published:** 2014-08-27

**Authors:** Bingjun Jiang, Haiyang Nan, Youfei Gao, Lili Tang, Yanlei Yue, Sijia Lu, Liming Ma, Dong Cao, Shi Sun, Jialin Wang, Cunxiang Wu, Xiaohui Yuan, Wensheng Hou, Fanjiang Kong, Tianfu Han, Baohui Liu

**Affiliations:** 1 The National Key Facility for Crop Gene Resources and Genetic Improvement and MOA Key Lab of Soybean Biology (Beijing), Institute of Crop Sciences, The Chinese Academy of Agricultural Sciences, Beijing, China; 2 The Key Laboratory of Soybean Molecular Design Breeding, Northeast Institute of Geography and Agroecology, Chinese Academy of Sciences, Harbin, China; 3 University of Chinese Academy of Sciences, Beijing, China; Institute of Genetics and Developmental Biology, Chinese Academy of Sciences, China

## Abstract

Soybean cultivars are extremely diverse in time to flowering and maturation as a result of various photoperiod sensitivities. The underlying molecular genetic mechanism is not fully clear, however, four maturity loci *E1, E2, E3* and *E4* have been molecularly identified. In this report, cultivars were selected with various photoperiod sensitivities from different ecological zones, which covered almost all maturity groups (MG) from MG 000 to MG VIII and MG X adapted from latitude N 18° to N 53°. They were planted in the field under natural daylength condition (ND) in Beijing, China or in pots under different photoperiod treatments. Maturity-related traits were then investigated. The four *E* maturity loci were genotyped at the molecular level. Our results suggested that these four *E* genes have different impacts on maturity and their allelic variations and combinations determine the diversification of soybean maturity and adaptation to different latitudes. The genetic mechanisms underlying photoperiod sensitivity and adaptation in wild soybean seemed unique from those in cultivated soybean. The allelic combinations and functional molecular markers for the four *E* loci will significantly assist molecular breeding towards high productivity.

## Introduction

Soybean (*Glycine max* (L.) Merrill) is a short-day crop with high protein and oil contents. Many cultivars were bred with different maturity to adapt to various ecological environments. For the convenience of breeding layout, 13 MGs from MG000 to MGX were classified in North America [1]–[3]. Chinese soybean researchers divided cultivars into different maturity groups as well [4]–[5]. Soybean production has increased in America in response to recent increases in global demand and maturity is the key factor determining soybean productivity. Therefore, it is rather important to understand the mechanism of soybean maturity diversity and adaptation.

Flowering and maturity were highly controlled by major genes in soybean. Up to now, nine maturity loci have been identified as *E1*–*E8* and *J* [1], [6]–[12]. These loci have different roles under different photoperiods. Wang et al [13] found that long daylength condition (LD) might strengthen while short daylength condition (SD) might weaken these maturity loci. More results and progress of maturity genes was reviewed by Xia et al [14]. Furthermore, four loci were identified at molecular level. *E1* gene was identified as a transcription factor which functions as a flowering repressor with a putative nuclear localization signal and a B3-related domain [15]. *E2* is an orthologue of Arabidopsis flowering gene *GIGANTEA* [16]. *E3* and *E4* are phytochrome genes *GmPhyA3* [17] and *GmPhyA2* [18], respectively. In addition, two homologs of soybean *Flowering Locus T* (*FT*) genes, *GmFT2A* and *GmFT5A* were identified and coordinately regulate flowering [19]. Four identified maturity genes *E1*, *E2*, *E3* and *E4* delay flowering and maturity under LD through down regulating *GmFT2A* and *GmFT5A* [15], [16], [19]. As for other loci, more studies should be done before learning their molecular identities.

Although these four known loci *E1*, *E2*, *E3* and *E4* provide an important key to learn the mechanism of flowering and maturity, we mainly got knowledge based on few cultivars but not on a population level. Population-level knowledge will provide another different view of these four loci’s role on maturity and adaptation. Therefore, in this study we selected a set of soybean cultivars which cover 12 maturity groups from MG000 to MGVIII and MGX plus some cultivars with wide range of latitude from N 18° to N 53°. These cultivars were subjected to different photoperiod treatments. Traits of beginning bloom (R1), physiological maturity (R7) and full maturity (R8) were investigated [20]. Maturity loci *E1*, *E2*, *E3* and *E4* were genotyped in the population. Further association analysis was done. The results showed that allelic combinations of these four *E* genes significantly determine the ecological-economical adaption of cultivars although they have different impacts on maturity. In addition, the genetic mechanisms underlying photoperiod sensitivity and adaptation in wild soybean seemed unique from those in cultivated soybean.

## Materials and Methods

Soybean cultivars were selected from North America (Table 1), China and Russia (Table 2) [21]–[23]. Four wild soybean accessions were also included for genotyping only (Table 2), which are CAAE087 (Heiheyesheng) collected in Heihe (N 50°22′, E 127°53′), Heilongjiang, China; CAAE088 (Bayanyesheng) in Bayan (N 46°08′, E 127°39′), Heilongjiang, China; CAAE089 (Baiyangdianyesheng) in Baoding (N 38°51′, E 115°30′), Hebei, China; and CAAE090 (Guangxiyesheng) in Nanning (N 22°48′, E 108°19′), Guangxi, China. They covered 12 maturity groups from MG 000 to MG VIII and MG X and ranged from N 18° to N 53° indicated in Table 1 and Table 2.

**Table 1:** Cultivars from North America and their respective maturity group.

Code	PI number	Variety	MG	Code	PI number	Variety	MG
**CAAE001**	PI548594	Maple Presto	000	**CAAE021**	PI534646	Flyer	IV
**CAAE002**	PI567787	OAC Vision	000	**CAAE022**	PI598222	TN4-94	IV
**CAAE003**	PI548648	Canatto	00	**CAAE023**	PI564849	Nathan	V
**CAAE005**	PI592523	Glacier	00	**CAAE024**	PI572239	Holladay	V
**CAAE006**	PI629004	MN0201	0	**CAAE025**	PI633609	Lonoke	V
**CAAE007**	PI596541	Traill	0	**CAAE026**	PI561400	Rhodes	V
**CAAE008**	PI612764	MN0901	0	**CAAE027**	PI633610	Desha	VI
**CAAE009**	PI599300	Surge	NA	**CAAE028**	PI592756	Dillon	VI
**CAAE010**	PI548641	Haroson	I	**CAAE029**	PI617045	NC-Roy	VI
**CAAE011**	PI614833	NE1900	I	**CAAE030**	PI599333	Musen	VI
**CAAE012**	PI608438	Titan	I	**CAAE031**	PI531068	Stonewall	VII
**CAAE013**	PI561858	Holt	II	**CAAE032**	PI595645	Benning	VII
**CAAE014**	PI567786	OAC Talbot	II	**CAAE033**	PI617041	Santee	VII
**CAAE015**	PI533655	Burlison	II	**CAAE034**	PI555453	Hagood	VII
**CAAE016**	PI595926	Athow	III	**CAAE035**	PI603953	Motte	VIII
**CAAE017**	PI548634	Zane	III	**CAAE036**	PI548970	Foster	VIII
**CAAE018**	PI593258	Macon	III	**CAAE037**	PI548663	Dowling	VIII
**CAAE019**	PI578057	Saline	III	**CAAE038**		Jupiter	X
**CAAE020**	PI614155	NS93-4118	IV				

NA, not available.

**Table 2:** Cultivars and accessions from China and far-east Russia and their adaption latitudes.

Code	Variety	Latitude	Code	Variety	Latitude
**CAAE039**	Dengke 2	N 47°–53°	**CAAE064**	Fengchengzaochadou	N 25°–30°
**CAAE040**	Huajiang 4	N 48°–50°	**CAAE065**	Jin 6606	N 41°–43°
**CAAE041**	Heihe 27	N 46°–48°	**CAAE066**	Jinzhou 8–14	N 40°–43°
**CAAE042**	Heihe 3	N 46°–50°	**CAAE071**	Bahong 1	N 39°–42°
**CAAE043**	Heihe 43	N 46°–48°	**CAAE072**	Mianyanghuangwofeng	N 30°
**CAAE044**	Suinong 14	N 44°–48°	**CAAE073**	Lüpidou	N 25°–28°
**CAAE045**	Hefeng 25	N 39°–43°	**CAAE074**	Liuyuezao	N 25°–28°
**CAAE047**	Jilin 3	N 44°–46°	**CAAE075**	Ruijinxiaohuangdou	N 24°–27°
**CAAE049**	Jiunong 21	N 42°–46°	**CAAE076**	Edou 2	N 30°–35°
**CAAE050**	Jilin 30	N 41°–43°	**CAAE077**	Yulindahuangdou	N 23°
**CAAE052**	Jindou 19	N 35°–40°	**CAAE081**	Qiudou 1	N 25°–30°
**CAAE053**	Tiefeng 31	N 35°–40°	**CAAE082**	Jiangledaqingdou	N 27°
**CAAE054**	Jidou 12	N 35°–38°	**CAAE084**	Guixia 1	N 22°–26°
**CAAE055**	Qihuang 28	N 34°–37°	**CAAE085**	Nandou 12	N 29°–32°
**CAAE056**	Zhonghuang 13	N 30°–40°	**CAAE086**	Zigongdongdou	N 29°
**CAAE057**	Xudou 9	N 32°–35°	CAAE087*	Heiheyesheng	N 50°
**CAAE058**	Xudou 1	N 32°–35°	CAAE088*	Bayanyesheng	N 46°
**CAAE059**	Fengshouhuang	N 35°–38°	CAAE089*	Baiyangdianyesheng	N 39°
**CAAE061**	Dandou 2	N 40°–42°	CAAE090*	Guangxiyesheng	N 23°
**CAAE062**	Yuejin 4	N 35°–38°	**CAAE091**	Mohe 1	N 53°
**CAAE063**	Jinda 814	NA	**CAAE092**	Ziweicika 4/75	N 50°

CAAE092 is a cultivar from far east Russia; *indicates wild soybeans: CAAE087, CAAE088, CAAE089 and CAAE090.

Due to seed availability, 59 cultivars were selected for both field and pot experiments, 12 cultivars only in the field experiment, and 4 cultivars only in the pot experiment. For the field experiment, seeds were sowed on May 14^th^, 2012 in Beijing (N 39°97′, E 116°34′) and maturity-related traits of R1, R7 and R8 were recorded regularly [20]. For the pot experiment, seeds were sowed in 10-liter pots on May 16^th^ 2012 and grown under ND in Beijing. After emergence, the seedlings were thinned until each pot contained five uniform plants. These uniform plants were grown until the unifoliate expanded then treated with different photoperiods (LD, 16 h light/8 h dark; SD, 12 h light/12 h dark; and ND). For SD, the plants were transferred to dark room to shorten the daylength. For LD, incandescent bulbs (50 µmol m^−2^ s^−2^ at the top of plants) with automatic timer controls were used to extend the daylength. Additional details of plant growth and treatments were the same as reported by Wu et al [24]. The days to first flowering of each plant was recorded. Both experiments finished on Oct 15th, 2012. Photoperiod sensitivity (PS) was thus calculated as the following function, where DFF_LD_ is the days to first flowering (R1) from the expansion of the first pair of unifoliates (V1) under LD while DFF_SD_ under SD [25].
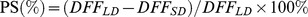



Genomic DNA was isolated from soybean unifoliate leaves using TianGen New Plant Genomic DNA Isolation Kit (DP320). Maturity loci *E1*, *E2*, *E3* and *E4* were genotyped using functional allele specific molecular markers [26].

### Ethics Statement

No specific permissions were required for domestic research of the collections of wild soybean accessions in Heihe (N 50°22′, E 127°53′), Bayan (N 46°08′, E 127°39′), Baoding (N 38°51′, E 115°30′) and Nanning (N 22°48′, E 108°19′), China. All the field studies did not involve endangered or protected species.

## Results

### Soybean cultivars have diverse flowering and maturity dates

**Figure 1:**
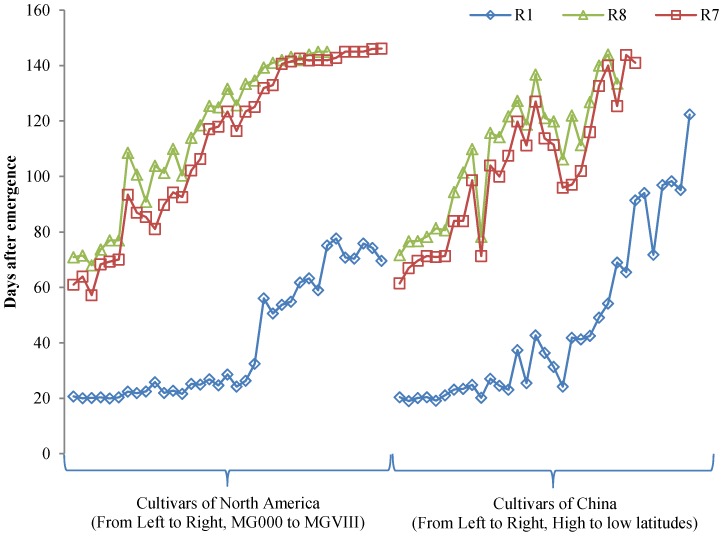
Maturity-related traits of soybean R1, R7 and R8 in the field at Beijing. Left, cultivars of North America, which are approximately sorted by maturity group. Right, cultivars of China, which are sorted roughly by adaption latitude.

**Figure 2:**
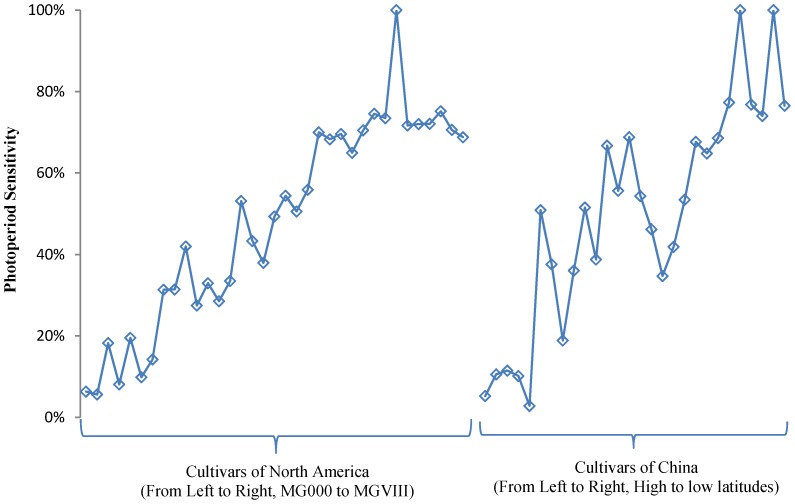
Photoperiod sensitivity of soybean cultivars. Left, cultivars of North America, which are approximately sorted by maturity group. Right: cultivars of China, which are sorted roughly by adaption latitude.

Seventy-one cultivars were planted under ND in the field at Beijing. These cultivars showed rich diversity in maturity (Table 3). Two cultivars CAAE081 and CAAE086 failed to flower until the experiment ended. Nine cultivars CAAE032, CAAE034, CAAE036, CAAE038, CAAE075, CAAE077, CAAE082, CAAE084 and CAAE085, flowered but did not reach R7. Six cultivars CAAE031, CAAE033, CAAE035, CAAE037, CAAE072 and CAAE076 did not reach R8 although they flowered and podded. For other cultivars that regularly flowered and matured, the days to R1 from emergence (VE) ranged from 19.0 (CAAE040) to 75.1 (CAAE030), the days to R7 ranged from 57.2 (CAAE003) to 142.5 (CAAE027), and the days to R8 ranged from 68.0 (CAAE003) to 145.0 (CAAE029 and CAAE030). Thus, the range of time to R1, R7 and R8 showed the maturity diversity of these cultivars. The cultivars that reached R1 less than 50 days after emergence could mature before the frost in Beijing, those that reached R1 in greater than 50 days but less than 70 days could mature partially, and the ones that reached R1 after 70 days could hardly mature (Table 3). In Figure 1 and Figure 2, maturity-related traits of R1, R7 and R8 generally increased from early MG to late MG and from high latitude to low latitude.

**Table 3:** The days from VE to R1, R7 and R8 of soybean Cultivars under ND condition at Beijing.

Code	Phenotype			Code	Phenotype		
	VE-R1	VE-R7	VE-R8		VE-R1	VE-R7	VE-R8
**CAAE001**	20.7±1.7	61.0±3.8	70.9±1.8	**CAAE038**	122.3±1.0	NA	NA
**CAAE002**	20.0±1.8	63.9±3.4	71.5±1.9	**CAAE039**	20.4±0.8	61.5±4.8	71.7±2.0
**CAAE003**	20.1±0.4	57.2±1.0	68.0±0.0	**CAAE040**	19.0±0.0	67.0±6.7	76.7±3.4
**CAAE005**	20.3±1.8	68.4±1.6	73.7±1.0	**CAAE041**	20.1±0.4	69.7±0.5	76.7±1.3
**CAAE006**	19.9±1.3	69.3±3.0	77.0±2.2	**CAAE042**	20.4±1.5	71.3±1.8	78.3±2.1
**CAAE007**	20.3±2.0	70.1±2.2	77±2.2	**CAAE043**	19.1±0.5	71.0±2.2	81.3±6.5
**CAAE008**	22.4±1.6	93.4±10.6	108.5±15	**CAAE044**	21.1±0.7	71.3±2.4	80.7±6.3
**CAAE009**	21.9±1.2	86.9±2.1	100.7±3.6	**CAAE045**	20.2±0.4	71.3±1.8	78.3±2.1
**CAAE010**	22.5±1.4	85.3±2.0	90.9±1.6	**CAAE047**	23.1±2.3	83.9±1.3	94.4±4.2
**CAAE011**	25.8±1.9	81.1±11.1	103.9±5.9	**CAAE049**	23.4±1.2	83.9±9.9	101.4±7.0
**CAAE012**	21.9±1.4	89.7±1.6	101.3±2.6	**CAAE050**	24.9±1.1	98.6±5.2	109.9±7.3
**CAAE013**	22.7±1.4	94.2±1.8	109.9±4.7	**CAAE052**	24.5±0.8	99.9±2.8	114.2±2.4
**CAAE014**	21.6±0.5	92.5±3.0	100.3±3.1	**CAAE053**	23.1±0.3	113.6±3.0	121.7±2.2
**CAAE015**	25.2±1.9	102.1±7.3	113.9±5.3	**CAAE054**	37.4±0.6	119.8±6.6	127.3±7.0
**CAAE016**	24.9±1.0	106.3±7.0	118.5±3.5	**CAAE055**	42.7±2.3	127.0±8.0	136.8±5.1
**CAAE017**	26.9±1.8	117.1±2.3	125.5±1.5	**CAAE056**	36.4±2.0	113.7±6.3	121.1±2.7
**CAAE018**	24.7±0.6	117.9±1.8	124.9±3.3	**CAAE057**	31.3±1.0	111.4±2.5	119.9±1.4
**CAAE019**	28.6±1.2	123.4±2.7	131.6±3.3	**CAAE058**	24.2±1.0	95.9±2.8	106.2±5.2
**CAAE020**	24.2±1.2	116.4±2.8	125.7±1.9	**CAAE059**	25.5±0.5	111.2±3.1	118.7±2.6
**CAAE021**	26.3±1.3	123.4±3.3	133.4±2.9	**CAAE063**	41.9±2.1	116.0±2.0	122.0±0.0
**CAAE022**	32.5±4.4	125.1±4.1	134.6±1.4	**CAAE064**	41.3±1.0	101.9±3.7	111.3±4.7
**CAAE023**	56.1±3.9	131.9±7.8	139.3±2.4	**CAAE065**	27.1±1.2	103.9±9.1	115.7±5.1
**CAAE024**	50.6±2.6	132.9±6.1	141.0±3.9	**CAAE066**	42.5±1.5	116.0±3.2	126.8±6.3
**CAAE025**	53.7±1.1	140.6±4.5	142±0.0†	**CAAE071**	49.1±4.0	132.6±6.4	140.0±5.6
**CAAE026**	54.9±1.9	141.5±4.4	143.2±1.8	**CAAE072**	91.3±2.7	141.0±1.4	NA
**CAAE027**	61.8±4.6	142.5±4.9	142.0±0.0†	**CAAE073**	54.2±1.1	140.0±4.7	144.0±2.8†
**CAAE028**	63.3±5.5	141.8±4.3	144.0±1.7†	**CAAE074**	69.0±0.0	125.4±4.6	133.5±4.6
**CAAE029**	59.0±5.2	142.0±3.1	145.0±0.0†	**CAAE075**	94.0±2.1	NA	NA
**CAAE030**	75.1±2.0	141.9±3.0	145.0±0.0†	**CAAE076**	65.5±5.5	143.8±1.6	NA
**CAAE031**	77.7±3.3	142.8±1.1	NA	**CAAE077**	71.8±9.8	NA	NA
**CAAE032**	70.8±3.2	NA	NA	**CAAE081**	NA	NA	NA
**CAAE033**	70.4±3.1	145.0±0.0	NA	**CAAE082**	96.8±1.8	NA	NA
**CAAE034**	77.7±1.6	NA	NA	**CAAE084**	98.3±2.9	NA	NA
**CAAE035**	75.9±4.4	145.0±0.0	NA	**CAAE085**	95.1±2.6	NA	NA
**CAAE036**	74.3±4.5	NA	NA	**CAAE086**	NA	NA	NA
**CAAE037**	69.6±4.6	146.1±0.9	NA				

† Partial plants (<50%) matured. NA, not available.

### Soybean cultivars react variously to photoperiod treatments

Sixty-three cultivars were planted in pots. They were treated with different photoperiods after V1. They began flowering in 18.4 to 32.8 days after emergence under SD, in 20.0 to 122.3 days under ND, and in 20.5 to 113.7 under LD, while under LD three cultivars CAAE031 (PI531068, MGVII), CAAE084 (Qiudou 1), and CAAE086 (Zigongdongdou) did not flower (Table 4). The PS was calculated according to the equation [25], which ranged between 10% and 80%. For the three cultivars that did not flower, it could be set at 100%. Thus, CAAE035 (PI603953, MGVIII), CAAE072 (Mianyanghuangwofeng), CAAE085 (Nandou 12), CAAE075 (Ruijinxiaohuangdou) and the former three cultivars are most sensitive to photoperiod (PS>75%). CAAE001 (PI548594, MG000), CAAE002 (PI567787, MG000), CAAE005 (PI592523, MG00), CAAE007 (PI596541, MG0), CAAE042 (Heihe 3) and CAAE091 (Mohe 1) could be classified as photoperiod insensitive because their photoperiod sensitivities are lower than 10%. These data suggested that these soybean cultivars diversify significantly in photoperiod sensitivity. Moreover, as shown in Figure 2, the photoperiod sensitivity of these cultivars generally increased from early MG to late MG and from high to low latitude.

**Table 4:** The days to R1 from V1 under different photoperiod treatments and the resultant PS at Beijing.

Cultivar	Days from V1 to R1			PS (%)	Cultivar	Days from V1 to R1			PS (%)
	SD	ND	LD			SD	ND	LD	
**CAAE001**	21.4±1.1	20.4±0.7	22.9±2.4	6.3	**CAAE035**	26.9±1.3	83.2±1.1	108.2±8.9	75.2
**CAAE002**	22.6±1.1	20.9±1.4	23.9±2.2	5.6	**CAAE036**	26.7±2.4	83.9±1.4	90.6±0.5	70.6
**CAAE003**	21.4±1.5	24.7±1.7	26.2±3.3	18.2	**CAAE037**	27.6±0.7	73.9±3.1	88.4±0.5	68.8
**CAAE005**	21.4±2.0	20.0±0.6	23.2±0.9	8.1	**CAAE050**	23.1±0.9	30.2±1.4	37.1±5.0	37.6
**CAAE006**	21.7±2.1	22.0±2.5	27.0±5.4	19.5	**CAAE053**	23.1±1.7	28.9±2.2	37.8±6.5	38.8
**CAAE007**	20.6±1.3	20.1±0.6	22.9±1.7	9.8	**CAAE052**	22.9±0.8	31.9±1.9	47.2±3.8	51.5
**CAAE008**	24.1±3.2	25.5±0.9	28.1±4.1	14.2	**CAAE054**	24.1±1.1	44.1±1.8	54.3±3.0	55.6
**CAAE009**	22.6±2.7	29.8±1.9	32.9±3.4	31.4	**CAAE057**	27.0±0.8	45.0±2.7	50.2±1.9	46.2
**CAAE010**	22.8±1.7	28.5±2.1	33.2±1.6	31.4	**CAAE056**	28.0±1.7	49.5±1.4	61.3±3.2	54.3
**CAAE011**	24.8±1.6	32.5±1.5	42.7±3.4	42.0	**CAAE061**	26.7±3.1	50.3±4.6	54.3±7.1	50.9
**CAAE012**	25.1±3.0	27.9±2.3	34.6±3.1	27.5	**CAAE076**	27.3±0.8	73.3±1.5	86.9±0.9	68.5
**CAAE013**	22.2±2.3	28.6±2.1	33.1±2.5	33.0	**CAAE064**	32.8±2.0	45.9±2.4	56.5±2.8	41.9
**CAAE014**	24.8±1.9	28.7±2.6	34.7±4.7	28.5	**CAAE059**	24.1±1.4	44.8±1.6	77.4±2.0	68.8
**CAAE015**	26.7±2.1	31.2±2.6	40.1±3.7	33.5	**CAAE084**	30.8±1.9	106.4±0.7	NA	100.0
**CAAE016**	22.0±1.9	31.2±3.2	46.9±5.2	53.1	**CAAE045**	24.3±2.1	27.6±1.5	30.0±2.6	18.9
**CAAE017**	26.2±2.1	35.6±6.0	46.3±0.5	43.4	**CAAE041**	21.2±1.7	22.8±0.9	23.6±1.3	10.2
**CAAE018**	25.1±0.4	31.5±0.8	40.5±1.6	38.0	**CAAE042**	23.0±1.7	24.3±1.4	23.7±1.7	2.8
**CAAE019**	25.3±2.1	39.4±1.0	49.9±3.1	49.3	**CAAE040**	20.2±1.5	20.4±0.5	22.8±1.6	11.5
**CAAE020**	21.3±1.6	34.1±6.0	46.7±4.2	54.4	**CAAE065**	26.4±1.4	34.1±2.9	41.3±2.7	36.0
**CAAE021**	23.7±2.7	36.7±3.1	47.9±3.6	50.5	**CAAE066**	26.5±0.5	48.6±1.3	56.9±1.9	53.4
**CAAE022**	23.2±2.2	45.2±2.5	52.5±5.0	55.9	**CAAE074**	32.2±1.0	62.9±0.3	91.5±0.9	64.8
**CAAE024**	25.0±0.8	60.2±1.2	83.3±0.6	70.0	**CAAE073**	26.6±1.2	63.8±5.1	82.0±0.7	67.6
**CAAE025**	27.9±1.4	67.1±1.4	87.9±1.1	68.3	**CAAE072**	28.2±1.8	92.7±3.3	124.4±0.8	77.3
**CAAE026**	26.4±1.3	67.2±0.8	86.9±0.8	69.6	**CAAE091**	20.4±2.8	22.9±0.7	21.6±1.2	5.2
**CAAE027**	29.5±1.0	67.0±0.5	84.0±0.0	64.9	**CAAE085**	29.8±1.7	98.6±1.3	126.8±1.7	76.5
**CAAE028**	26.3±2.0	68.1±1.3	89.0±2.3	70.5	**CAAE075**	28.9±1.4	98.3±1.4	124.5±1.2	76.8
**CAAE029**	24.1±0.8	82.1±3.3	94.7±0.5	74.5	**CAAE058**	23.9±1.4	30.9±1.9	36.6±7.0	34.7
**CAAE030**	25.0±0.8	83.8±1.4	94.1±0.6	73.4	**CAAE077**	29.6±2.8	96.4±0.5	113.7±1.0	74.0
**CAAE031**	24.7±0.8	83.6±0.5	NA	100.0	**CAAE062**	27.6±0.9	55.3±2.3	83.0±1.4	66.7
**CAAE032**	25.1±0.7	72.6±1.3	88.6±1.1	71.7	**CAAE092**	18.4±2.0	20.9±1.6	20.5±1.8	10.6
**CAAE033**	24.9±0.4	73.9±2.2	88.9±1.9	72.0	**CAAE086**	32.8±0.8	122.3±0.8	NA	100.0
**CAAE034**	24.9±0.6	84.6±1.1	89.0±1.7	72.0					

NA, not available.

### Genotyping soybean cultivars of maturity loci *E1*, *E2*, *E3* and *E4*

Eighty-five cultivars were genotyped at four maturity loci *E1*, *E2*, *E3* and *E4* (Table 4). Thirty-eight cultivars are from North America, which cover 12 maturity groups from MG000 to MGVIII and MGX. Other cultivars are from China except CAAE092, which is from Russia (Tables 1 and 2). There are ten genotypes in total in this population (Table 5). The genotypes of *E1/E2/E3/E4* and *E1/e2/E3/E4* are the majority types, which were identified in 28 and 19 cultivars respectively (Table 5). Three genotypes of *e1-as/E2/e3/E4*, *e1-as/e2/e3/e4* and *e1/e2/e3/E4* were identified only in one variety each (Table 5). For the *E1* locus, allele *e1* and *el-as* are always detected in early-maturing cultivars from MG000 to MGIV or from high latitudes adapted cultivars. Moreover, all of the four wild soybeans were *E1/E2/E3/E4* type.

**Table 5:** Genotype of soybean cultivars at four maturity loci *E1*, *E2*, *E3* and *E4.*

Genotype				Num	Variety
E1	E2	E3	E4		
*E1*	*E2*	*E3*	*E4*	28	CAAE023 (MGV), CAAE024 (MGV), CAAE025 (MGV), CAAE026 (MGV),CAAE027 (MGVI), CAAE028 (MGVI), CAAE029 (MGVI), CAAE030(MGVI), CAAE031 (MGVII), CAAE032 (MGVII), CAAE033 (MGVII),CAAE034 (MGVII), CAAE035 (MG VIII), CAAE036 (MG VIII), CAAE037(MG VIII), CAAE038 (MGX), CAAE071, CAAE072, CAAE075,CAAE081, CAAE082, CAAE084, CAAE085, CAAE086, CAAE087,CAAE088, CAAE089, CAAE090
*E1*	*e2*	*E3*	*E4*	19	CAAE047, CAAE049, CAAE050, CAAE052, CAAE054, CAAE055,CAAE056, CAAE057, CAAE058, CAAE061, CAAE062, CAAE063,CAAE064, CAAE065, CAAE066, CAAE073, CAAE074, CAAE076,CAAE077
*E1*	*e2*	*e3*	*E4*	3	CAAE044, CAAE045, CAAE059
*e1-as*	*E2*	*E3*	*E4*	8	CAAE016 (MGIII), CAAE017 (MGIII), CAAE018 (MGIII),CAAE019 (MGIII), CAAE020 (MGIV), CAAE021 (MGIV),CAAE022 (MGIV), CAAE053
*e1-as*	*E2*	*e3*	*E4*	1	CAAE011 (MGI)
*e1-as*	*e2*	*E3*	*E4*	8	CAAE003 (MG00), CAAE008 (MG0), CAAE009, CAAE010(MGI), CAAE012 (MGI), CAAE013 (MGII), CAAE014(MGII), CAAE015 (MGII)
*e1-as*	*e2*	*e3*	*E4*	7	CAAE005 (MG00), CAAE006 (MG0), CAAE007(MG0), CAAE040, CAAE041, CAAE042,CAAE043
*e1-as*	*e2*	*e3*	*e4*	1	CAAE039
*e1*	*e2*	*e3*	*E4*	1	CAAE092
*e1*	*e2*	*e3*	*e4*	3	CAAE001 (MG000), CAAE002 (MG000),CAAE091

### Maturity loci *E1*, *E2, E3* and *E4* have different impacts on maturity and photoperiod response

**Figure 3:**
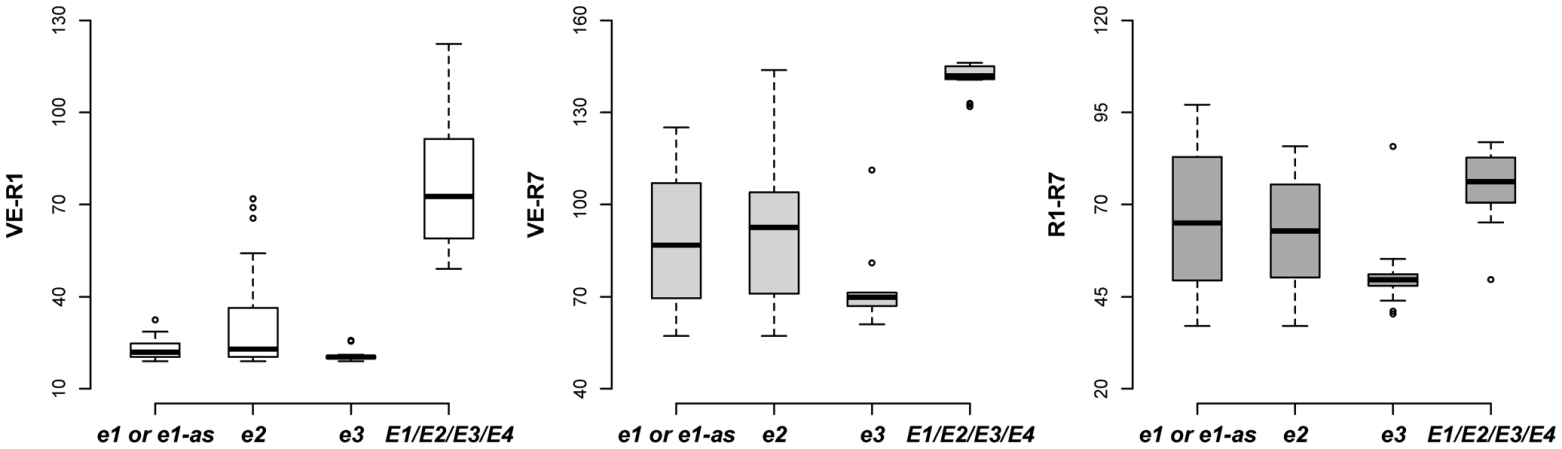
Quartile box plots showing days between the stages of VE, R1 and R7. Circles show outliers.

**Figure 4:**
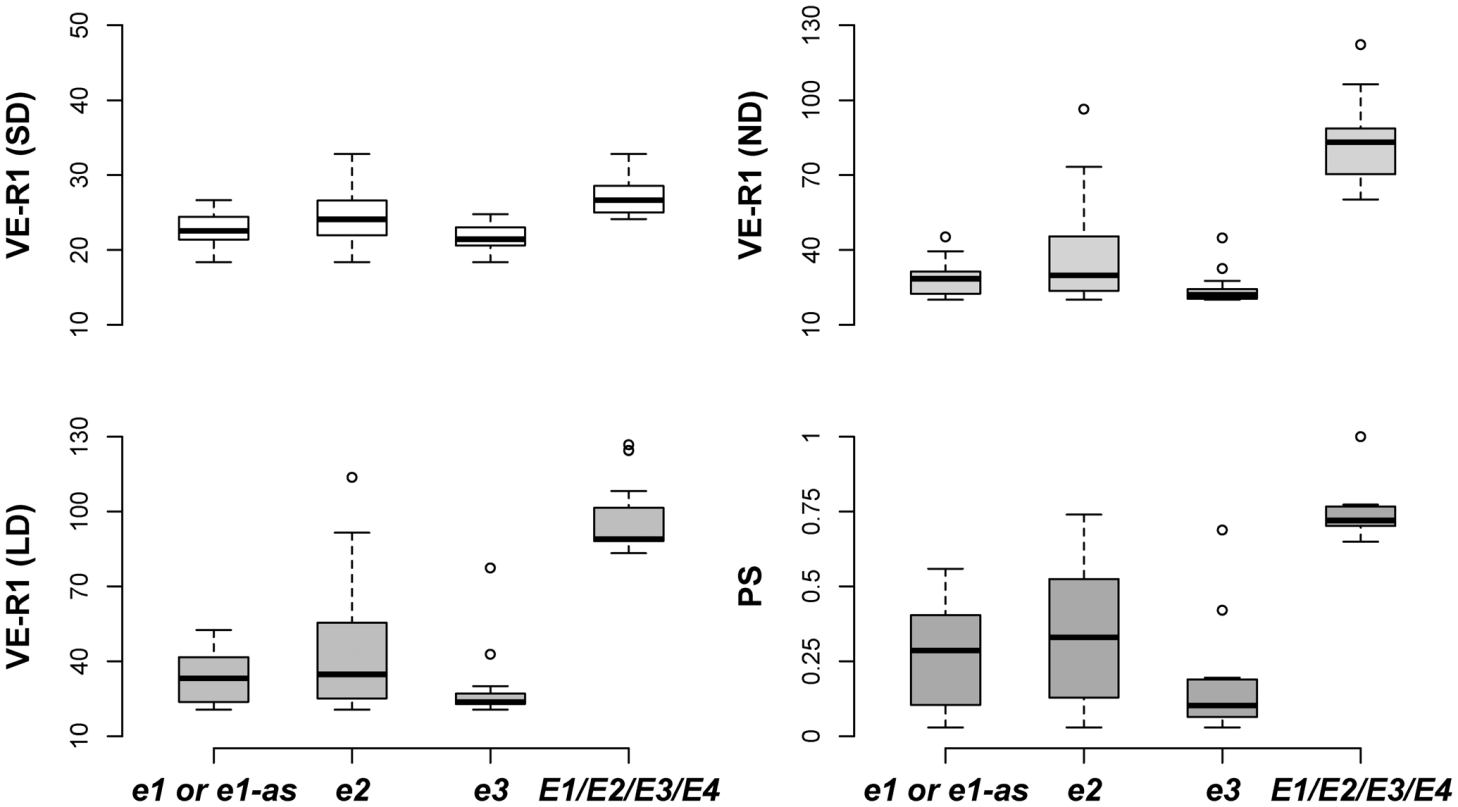
Quartile box plots showing days between the stages of VE and R1 under different photoperiod conditions and PS. Circles show outliers.

**Figure 5:**
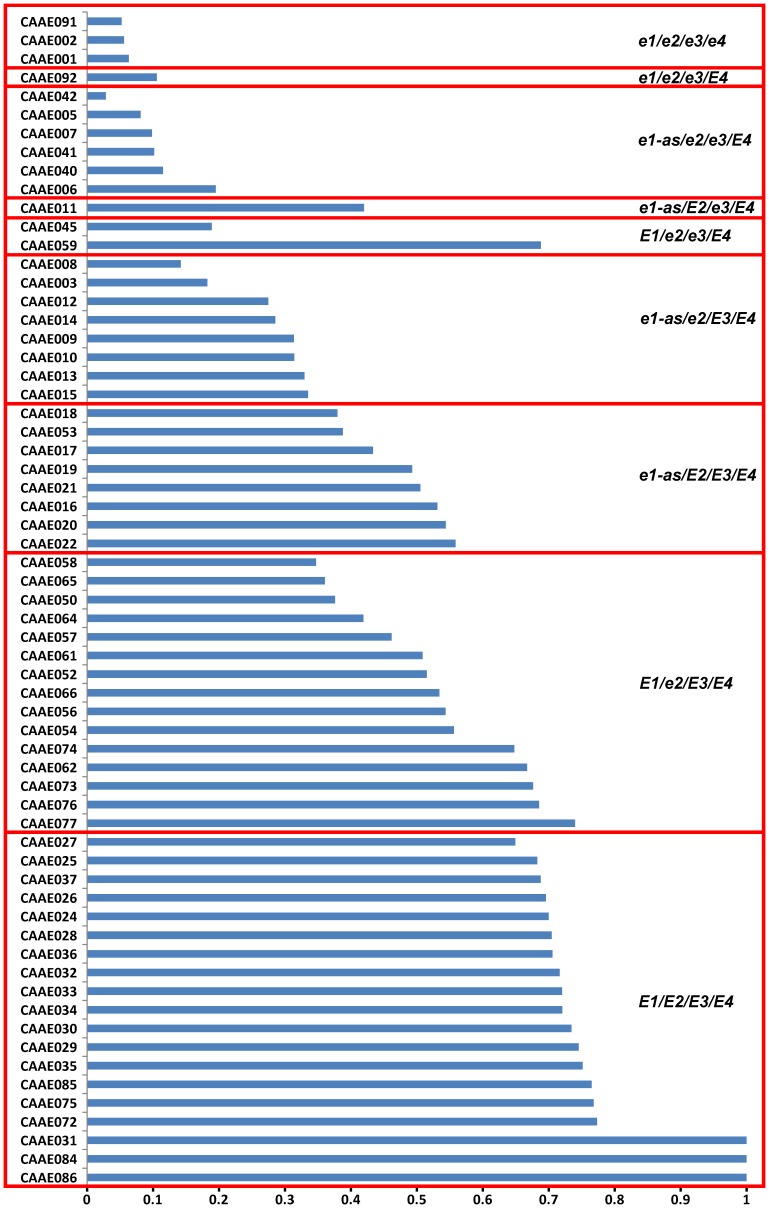
Photoperiod sensitivity grouped by *E* genotypes.

In general, recessive alleles *e1*, *el-as*, *e2*, *e3* and *e4* promoted flowering and maturity but with different impacts (Figure 3). The allele *e4* was detected in only four cultivars (CAAE039 with the genotype *e1-as/e2/e3/e4*, and CAAE001, CAAE002 and CAAE091 with the genotype of *e1/e2/e3/e4*) in the population. The four cultivars with recessive *e4* alleles were adapted to high latitude and showed photoperiod insensitivity suggesting the importance of the *e4* allele for high latitude adaptation. The cultivars with the allele *e1*, *e1-as* or *e2* exhibited a narrower range of the days from VE to R1 (VE-R1) than that of the days from VE to R7 (VE-R7) and that of the days from R1 to R7 (R1–R7) (Figure 3). In contrast, the cultivars with the allele *e3* or *e4* showed a consistently narrow range although some outliers existed (Figure 3). Moreover, these four recessive alleles promoted flowering under different photoperiod conditions (Figure 4), and the cultivars with more recessive alleles of *e1*, *e1-as*, *e2*, *e3* and *e4* had a lower PS during photoperiod treatments (Figure 5).

## Discussion

### Soybean cultivars from different maturity groups show diversity in flowering, maturity and photoperiod sensitivity

The tested soybean cultivars were selected from North America, China and Russia. Some of them covered from MG000 to MGVIII and MGX, almost all of the total 13 MG [2] and the others were collected from N 18° to N 53° to cover the wide range of latitude, which represents the main soybean producing area in China. In addition, some wild soybean accessions were also included. Thus, the population of lines used here should exhibit the diversity of maturity not only in phenotype but also in genotype. The field experiment under ND provided strong evidence. Some cultivars failed to flower (R1), some could not reach pod yellowing (R7) and some could not reach full maturity (R8) (Table 3). Even for those cultivars that flowered and matured, the days to first flowering, physiological maturity and full maturity varied significantly (Table 3). What’s more, in the experiment of photoperiod treatments, the population also showed diversity of photoperiod sensitivity as expected. The days to first flowering varied between different photoperiod treatments and between different cultivars. The photoperiod sensitivity also varied from 10% to 80% (Table 4). Six cultivars were classified as photoperiod insensitive: CAAE001 (PI548594, MG000, *e1/e2/e3/e4*), CAAE002 (PI567787, MG000, *e1/e2/e3/e4*), CAAE005 (PI592523, MG00, *e1-as/e2/e3/E4*), CAAE007 (PI596541, MG0, *e1-as/e2/e3/E4*), CAAE042 (Heihe 3, *e1-as/e2/e3/E4*) and CAAE091 (Mohe 1, *e1/e2/e3/e4*). These results were consistent with previous report that soybean photoperiod insensitivity was at least conditioned by three genetic mechanisms according to allelic combinations of *E1*, *E2* and *E4*: *e3/e4*; *e1/e3 or e1/e4* and *e1-as/e3/E4*. In the genetic mechanism of *e1-as/e3/E4,* novel unidentified gene/genes participated in photoperiod insensitivity [26], [27].

### *E* genes have different impacts on flowering and maturation

Soybean, as a short-day crop, has many cultivars with diversified maturity structure. *E1*, *E2* and *E3* are involved with different impacts. In the field experiment under ND, the recessive allele *e1*, *el-as* and *e2* significantly narrowed the variation of VE-R1 more than that of VE-R7 and R1–R7 (Figure 3), suggesting that *E1* and *E2* genes have significant impact on pre-flowering development other than post-flowering responses. However, the loci *E3* and *E4* might function not only in pre-flowering development but also in post-flowering development, indicated by the narrow variation in VE-R1, VE-R7 and R1–R7 of cultivars with *e3* and e4 alleles (Figure 3). This result is consistent with Xu et al [26]. that *E3* and *E4* respond not only to pre-flowering but also to post-flowering by increasing pod filling duration, number of nods and pod numbers by up-regulating the expression of growth habit gene *Dt1*. This result implies the significance of *E3* and *E4* loci for molecular genetic breeding to increase soybean productivity. Moreover, the outliers suggested that the disfunction of *E3* and *E4* might be interrupted by other genes. Similarly, the loci *E1* and *E3* are related with photoperiod sensitivity (Figure 4).

For the genotype of *E1/E2/E3/E4*, the photoperiod sensitivity was mostly above 70%. Compared with genotypes *E1/E2/E3/E4*, *e1-as/E2/E3/E4*, *e1-as/e2/E3/E4* and *e1-as/e2/e3/E4* (Figure 5), the photoperiod sensitivity decreased with the numbers of recessive alleles. While comparing *E1/e2/E3/E4* and *e1-as/E2/E3/E4* with *E1E2E3E4*, it was suggested that *E1* plays a more important role than *E2* because *e1* decreased the photoperiod sensitivity more significantly and narrowed its range. These results further proved that soybean photoperiod insensitivity was involved by four maturity loci *E1, E3, E4* and *E7* while *E2* locus was not involved [28], [29]. In these tested cultivars of China, *E1/e2/E3/E4* is much more abundant. It ranged from N 18° -N 42° while *E1/E2/E3/E4* was distributed south of N 39° except for the four wild soybeans (CAAE087, Heiheyesheng; CAAE088, Bayanyesheng; CAAE089, Baiyangdianyesheng; and CAAE090, Guangxiyesheng). For *e1* or *el-as* alleles, the associated cultivars where mostly located above N 45° except for CAAE053 (Tiefeng 31, N 35°–40°). Thus, to improve the ecological adaptability of cultivars, the *E1* gene must function less because of its most strong impact on delaying maturity while other *E* genes might become important in adaptation. Unlike cultivated soybean, two wild soybean accessions, CAAE087 (Heiheyesheng) and CAAE088 (Bayanyesheng) adapted north of N 46° in China where cultivars generally had less photoperiod sensitivity where genotyped as *E1/E2/E3/E4*. It is greatly important for wild soybean to adapt and survive during season alteration. The genetic mechanisms underlying photoperiod insensitivity and adaptation in wild soybean were therefore unique from those in cultivated soybean.

### Allelic combinations of *E* genes determine maturity groups

In the population analyzed here, MGV to MGX have the same genotype at *E1/E2/E3/E4*. It suggests that in these maturity groups, other unknown maturity genes should be involved in the determination of Mature Group. MGIII to MGIV are mainly genotyped as *e1-as/E2/E3/E4*, and MGII is *e1-as/e2/E3/E4*. For MGII to MGX, each group has one genotype. MGI has the genotypes of *e1-as/e2/E3/E4* and *e1-as/E2/e3/E4*; MG0 and MG00 both have the genotypes of *e1-as/e2/E3/E4* and *e1-as/e2/e3/E4*; and MG000 has *e1/e2/e3/e4.* From MG000 to MGI, each maturity group has two genotypes, which means that photoperiod is the first key factor in these region to determine maturity group. However, the more recessive alleles at *E* genes, the earlier cultivars mature. Due to the limited number of cultivars used in each MG, the maturity genotypes for each group may be underestimated and additional genotypes for each MG may be identified with a larger sample of cultivars. Although it is not possible to enumerate all genotypes of a given maturity group, this limited sample of cultivars showed that allelic combinations of *E* genes determine maturity groups in general.

## Conclusions

The *E* genes (*E1*, *E2*, *E3* and *E4*) have different roles in maturity and photoperiod sensitivity and their allelic combinations determine maturity group and adaptation to different latitude.
